# Music Is Life—Follow-Up Qualitative Study on Parental Experiences of Creative Music Therapy in the Neonatal Period

**DOI:** 10.3390/ijerph18126678

**Published:** 2021-06-21

**Authors:** Friederike Barbara Haslbeck, Lars Schmidli, Hans Ulrich Bucher, Dirk Bassler

**Affiliations:** Department of Neonatology, University Hospital Zurich, University of Zurich, 8091 Zurich, Switzerland; lars.schmidli@uzh.ch (L.S.); buh@usz.ch (H.U.B.); dirk.bassler@usz.ch (D.B.)

**Keywords:** extremely preterm infant, parents, creative music therapy, long-term development, daily life, qualitative interview, parental perspective, relaxation

## Abstract

Developmental problems in extremely preterm (EP) infants and the associated longitudinal burden for their families are major health issues worldwide. Approaches to social-emotional support such as family-integrating Creative Music Therapy (CMT) are warranted. We aimed: (1) to explore parental perspectives on the use of CMT with EP infants in the neonatal hospitalization period and (2) to examine the possible longitudinal influence of CMT. A qualitative design was used to examine the perspective of six families from various backgrounds. Semi-structured interviews were carried out when the infants reached school age. We used an inductive–deductive thematic analysis to identify three main themes, each with three sub-themes: (1) the positive impact of CMT on the infants, the parents, and bonding; (2) the attitude toward CMT, from being open-minded to recommending it as complementary therapy; and (3) the experience of overall healthy infant development despite unique developmental delay issues. The findings elucidate the positive and formative impact of CMT on both infants and parents in the stressful NICU setting and beyond. CMT may empower positive transformation in the parents through individualized early nurturing musical interactions, capacity building, and positive reinforcement. Further research may help to identify and implement potentially modifiable factors for improving health care in this vulnerable group through early family-integrating, resource-based approaches such as CMT.

## 1. Introduction

Developmental problems in preterm infants and the associated longitudinal burden for their families are major health issues worldwide [1]. In particular, extremely preterm (EP) infants (born at less than 28 weeks of gestational age) who are very immature suffer from short and long-term impairments. Although technological advances in medicine have improved the survival and outcomes of EP infants in recent decades, a reasonable risk for death (30–50% mortality) and disability (20–50%) remains [2,3,4]. Cognitive [5], behavioral, mental health [6], and learning disabilities [7] are common long-term problems in EP infants while the prevalence of severe disabilities, such as cerebral palsy, is decreasing [8].

The birth of a preterm infant is usually a stressful and traumatizing event for the parents [9]. Particularly, parents of extremely preterm infants face concerns about their infant’s survival and suffering and may be traumatized by the appearance of their infant with the innumerable tubes and wires in the unfamiliar, high-tech environment of the neonatal intensive care unit (NICU) [10]. Parents of EP infants are confronted with a tremendous shift in their expectations as to the parental role and can experience feelings of helplessness, shame, guilt, and anxiety, which may result in acute stress or posttraumatic stress disorders [11]. After hospital discharge, they often feel abandoned to deal with the further emotional, physical, and financial challenges of caring for their extremely prematurely born infant [12]. They may suffer from family functioning issues, psychological distress, depressive symptoms, and anxiety for months or even years after discharge [13], sometimes until their children reach school age [14].

Therefore, preventive approaches offering social-emotional, family-centered support are warranted for this vulnerable population. Besides various psychological and parental education nursing interventions in neonatal care [15], family-integrating music therapy interventions address both the physiological and psychological needs of infants and parents by focusing on parental empowerment and coping rather than on parental education [16,17,18]. Family-integrating CMT in particular is already provided to extremely preterm infants and their parents, starting with soft, calm, individually tailored humming in a lullaby style to stabilize, relax, and regulate using finely attuned musical parameters and therapeutic responsiveness [19,20]. Throughout the hospitalization trajectory, CMT is continuously adapted to the infants’ and parents’ needs to stimulate and interact with the infant and encourage parents to engage in vocal interactions. CMT uses meaningful, culturally sensitive songs and music of the family as well as song-writing to express parental feelings and empower the music-cultural identity of each family [21]. Parents are individually integrated into the therapeutic process, i.e., by providing music therapy during skin-to-skin contact to foster bi-directional relaxation, attachment, and bonding. Since CMT is based on the Nordoff–Robbins Music Therapy theories and methods, the core tenet is that almost every human being responds to music—no matter how premature, ill, or disabled [22].

Our recent prospective, randomized, controlled pilot trial examining functional magnet resonance imaging at term-equivalent age revealed that infants who received CMT at a Swiss NICU showed more reliable functional networks, higher functional integration, and shorter thalamocortical processing delays in several regions (left prefrontal, supplementary motor, inferior temporal) compared to infants in the control group [23]. These promising results suggest a potentially positive impact on long-term neurodevelopment as these brain regions are associated with discriminating sounds, language, cognition, fine motor coordination, and empathy [24,25]. The secondary outcomes of the study indicate that anxiety levels and depressive symptoms decreased while bonding increased in the parents of the CMT group compared to the control group [26]. The qualitative interviews shortly after discharge showed that CMT facilitated parental relaxation and joy and intensified infant-parent interactions. These results are in line with quantitative results of a meta-analysis on immediate beneficial effects of music therapy on maternal anxiety [27]. Accordingly, short-term qualitative results from various music therapy researchers confirm that music therapy may promote parental relaxation, well-being, and bonding in the neonatal period [28,29,30].

However, to our knowledge, there are no studies to date exploring the retrospective long-term parental perspective on music therapy with EP infants and its possible influence on their attitude toward music (therapy) in their daily life. In the present study, qualitative interviews were carried out with parents of formerly extremely preterm infants who have reached school age to add insights into the parental perspective and experience of CMT with these very fragile infants.

## 2. Materials and Methods

### 2.1. Study Design and Participants

We performed qualitative interviews with parents who participated in a controlled prospective, longitudinal, clinical feasibility trial with infants receiving CMT during their hospitalization in the Department of Neonatology of the University Hospital Zurich, Switzerland, around seven years ago (for details on the feasibility trial of the randomized controlled trial see: [31]. The participants were parents of the 13 EP infants consecutively recruited for the trial in the NICU (level-3 perinatal open unit center) from September 2013 to April 2014. Parents of preterm infants with the following criteria were eligible for inclusion: gestational age < 29 weeks, chronological age ≥ 7 days of life at the start of CMT, clinically stable requiring no invasive cardiorespiratory support. Preterm infants were excluded if they were diagnosed with a major congenital anomaly/genetically defined syndrome, congenital malformation adversely affecting life expectancy/neurodevelopment, intraventricular hemorrhage (≥Volpe grade 3) [32]. Further exclusion criteria were admission for palliative care or insufficient parental knowledge of the German language. Approval was given by the Ethics Committee of Canton Zurich, Switzerland, for the prospective feasibility trial (KEK-ZH_2013-0270) and amended qualitative inquiry (BASEC-NR. PB_2016-02589).

With a qualitative design, we aimed to explore and add insights into how parents experienced CMT in the NICU and beyond, including its relevance and personal meaning [33]. We used a qualitative, dual deductive–inductive approach [34] toward realist methods to investigate parental experiences and realities [35,36] and to elicit more tacit meanings [37]. In line with Ryan & Bernard [38], we aimed to identify overall themes that represent recurrent unifying patterns of meaning in the data set while characterizing specific parental experiences using thematic analysis [39].

### 2.2. Intervention

According to the clinical CMT protocol [19] the included infants and their parents received CMT for approximately 20 min two to three times per week until discharge. A qualified music therapist, the first author (FH), provided CMT at the bedside (incubator or warmer) following feeding time, either with the infant alone or with the parents conducting skin-to-skin contact. During CMT with the infant alone, the music therapist would hum smoothly and tailor vocalizations in line with the infant’s breathing rhythm, facial expression, gesture, and behavioral state. For example, the therapist would use sedating musical parameters (e.g., calm, repetitive humming) to soothe an agitated infant. Conversely, the therapist would use activating musical parameters (up-rising melodies) to stimulate a limp infant.

During the sessions with the parents, the parents hold their infant, providing skin-to-skin contact throughout the session. In some sessions, the music therapist placed an arched NICU-monochord with 29 strings (wooden vibro-acoustic string instrument provided by Saitenklang, Bern, Switzerland©) at the parents’ elbow to transmit relaxing vibrations via bone and air conduction. The music therapist then hums/sings in harmony with the monochord sounds and uses the same method of humming and singing, including the parents’ favorite songs and musical preferences. Throughout the music therapy process, parents are encouraged and empowered to respond to the infant’s cues and needs, as well as to hum and speak “motherese” [40] to facilitate bonding and attachment—see clinical CMT protocol for details [19].

The families received between 11 and 34 CMT sessions (mean (SD) total CMT sessions: 24.29 (±7.22)) for around twenty minutes two to three times a week during hospitalization. Per family, 10 to 27 sessions were conducted at the bedside with the infant alone (mean (SD) CMT session alone: 21.14 (±5.22)) and one to eight CMT sessions were conducted during skin-to-skin care together with the parents (and siblings) (mean (SD) CMT family sessions: 3.14 (±2.59)). No adverse reactions were observed during and directly after CMT at all, and it was clinically feasible to provide the intervention.

### 2.3. Data Collection

After the follow-up examination at five years, we contacted all feasibility study parents on their cell phone via text message (SMS) and invited them to engage in our qualitative follow-up inquiry. All included parents received an interview request for approximately half an hour, including written information and informed consent. The interview was conducted via telephone or face-to-face at the family home with solely the parents present. An independent researcher (the second author, a master student in medicine (LS)), who was not involved in the previous trial and the CMT interventions, conducted the interviews to avoid parental dependencies when answering the questions and allow for the inductive part of the dual deductive–inductive approach. Before assisting in the study, he was not familiar with CMT, nor with the families. He designed, conducted, and analyzed the interviews in close dialogue with the research team and supervisor (FH). The latter, as the inventor of CMT, was familiar with its methods and theories and represented the deductive part of the dual deductive-inductive approach. The second author pilot-tested the semi-structured, open-ended interview (Appendix A) with a random mother and audiotaped (iPad voice memo program) interviews for further transcription. He made memos of personal impressions and field notes of contextual details and non-verbal expressions after the interviews.

### 2.4. Data Analysis

The data analysis was conducted in an ongoing, iterative process from the early stages of data collection throughout data analysis to the reporting stages of the study [36], following the six phases of familiarization, generating codes, constructing themes, revising, and defining themes, and producing the report [39]. The process was realized in constant dialogue between the first and second author, and triangulated by discussions with other students to ensure the trustworthiness of the data analysis.

In the first phase, the second author familiarized himself with the data by reading the field notes and listening to the whole interview. He transcribed the interview data into an orthographic written form with verbal and nonverbal expressions (Appendix B). Audio files were transcribed manually, using dictation tools of Microsoft Word and “Anytune” (https://de.anytune.us/ (accessed on 15 November 2020)) to decelerate audio data. In this in-depth process of reading, re-speaking, and repeating the interview quotes, he already took first notes. He marked ideas that seemed particularly interesting concerning CMT and the further development of the infant.

In the second phase, the transcriptions were transferred to MaxQDA, a software program for qualitative research analysis (https://www.maxqda.com/ (accessed on 3 November 2020)). Transcripts were coded by tagging and naming selections of the interview quotes within each data item by identifying repeated patterns and meaning (Appendix B). The analysis was characterized by continuous navigation between codes of one and several interviews, the new data extracts and codes, and the entire data set [41].

**Figure 1:**
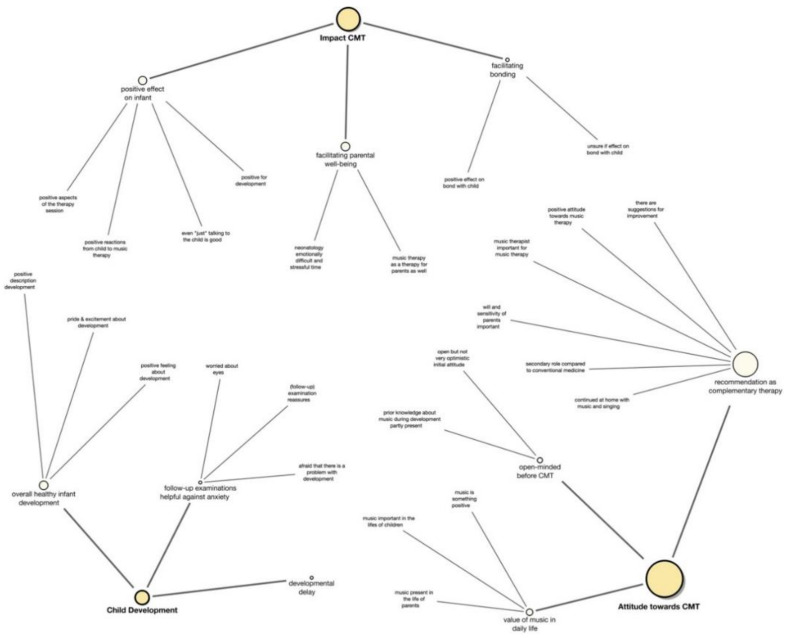
Final Coding Tree.

**Figure 2:**
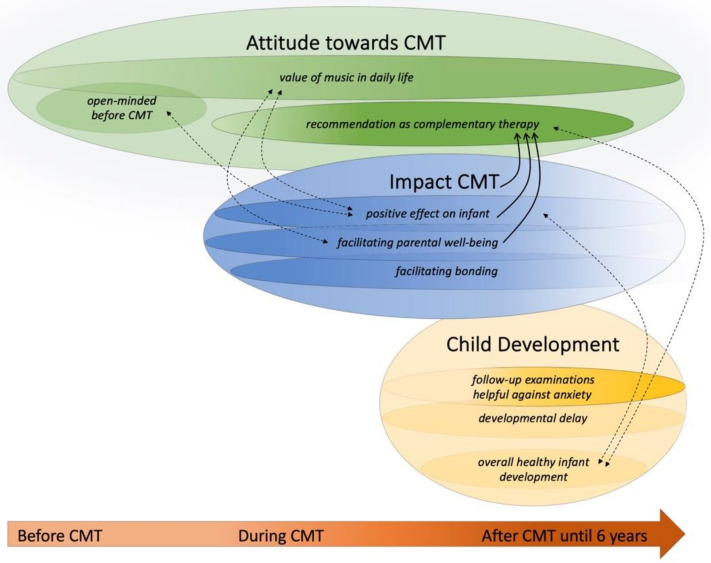
Final Thematic Map. Graphic Visualization of the Three Themes and the Nine Sub-Themes.

In the third phase, the second author analyzed and synthesized the codes to generate overarching preliminary sub-themes and produced a detailed coding tree (Appendix C). In the fourth and fifth phase, he reviewed all the themes relating to the coded data extracts and the whole data set to define and name coherent, consistent, and distinctive themes that seemed of particular value concerning the overall research question. The sub-themes were grouped into final themes and reviewed and refined throughout the sixth phase of producing the report, resulting in the final coding tree (Figure 1) and final thematic map (Figure 2) [39].

Overall coding tree (created with MAXQDA 2020 program) with the three themes (yellow circles), each theme’s three sub-themes (off-white circles), and codes (no circles); the size of the circles correlates to the number of coded segments they contain. The coding tree shows the inductive approach through which sub-themes emerged from different codes and themes from three different sub-themes (and codes from sub-codes as shown in Appendix C).

## 3. Results

### 3.1. Sample and Interview Characteristics

In November 2020, the ten families included in the feasibility clinical trial (September 2013–April 2014) were contacted via SMS for the interview request. Three families were unreachable, and one family declined to record the interview, so a final sample of six families with seven extremely preterm infants (60%) remained for the qualitative inquiry (Table 1). From mid-November to early December, five interviews were conducted via telephone and one interview at the family’s home face-to-face since this family explicitly requested an interview at home. Four mothers and one father were available for the telephone interviews. During the face-to-face interview with the couple, mainly the father spoke. The second author interviewed one mother in English, one father mainly in Swiss German, and the other four interviewees in High German. Interviews lasted around 30 min (range: 16–41 min).

The seven infants of the final sample were born at between 24 and 27 weeks of gestation (median 25 5/7), weighing between 610 and 1070 g at birth (median 808.5), all diagnosed with sepsis and necrotizing enterocolitis, two of them with retinopathy of prematurity, one with bronchopulmonary dysplasia, two twins and two boys, with a hospital stay of between 58 and 117 days (median 89) (Table 1).

The parents displayed a wide variety of cultural and socio-economic backgrounds. Two interview partners came from Switzerland; the others were from Slovenia, Kosovo, Denmark, and Germany. Three interview partners had completed a university degree, two apprenticeships, and one compulsory education. Two interview partners were already parents before giving birth to their preterm infant, whereas four of the mothers were primiparous (Table 1).

### 3.2. Findings

Three themes, each with three sub-themes, elucidating the meaning and experience of CMT and the infant development, were identified from the interview data (Figure 2):

1. Impact CMT: (a) positive effect on infant (b) facilitating parental wellbeing (c) facilitating bonding
2. Attitude toward CMT: (a) open-minded before CMT (b) value of music in daily life (c) recommendation as a complementary therapy
3. Child development: (a) developmental delay (b) follow-up examinations helpful against anxiety (c) overall healthy infant development

The parental experiences, challenges, attitudes, and perspectives developed and slightly changed over time by interacting and mutually influencing each other, as demonstrated in the thematic map by the longitudinal timeline, overlapping ovals, pointing arrows, and dashed lines (Figure 2). For example, before study recruitment, the general parental view of and attitude toward CMT was open-minded but not explicitly positive (subtheme 2a: openminded attitude before CMT). However, through the experienced positive impact of CMT on their infants and themselves (three sub-themes of theme 1: (a) positive effect on infant (b) facilitating parental wellbeing (c) facilitating bonding) the parental view developed toward a positive to even enthusiastic attitude. Finally, seven years later, the parental attitude, continuously influenced by the overall healthy development of their infant (sub-theme 3 c: overall healthy infant development) and the continuing significance of music in daily family life (subtheme 2b: the value of music in daily life) culminate in parental recommendations to provide CMT for all infants and their parents (subtheme 2c: recommendation as complementary therapy).

Moreover, the main theme impact of CMT was predominant in the interviews and is interrelated with most subthemes and themes. This was valued as most important by the data analyst (LS) and is presented as the central blue oval with the boldest black arrows in the graphic presentation (Figure 1). The positive effect on the parents themselves, who described how relaxing and supportive the therapy was in the stressful time of hospitalization, contributes to this most valuable theme of the inquiry. Interestingly, all parents contributed to the main and sub-themes, but the extent and trajectory varied individually. For example, all parents recommend CMT as a valuable complementary therapy (subtheme 2c). However, some parents were skeptical at the beginning and were positively surprised about the favorable effect of CMT, while others had anticipated a beneficial effect from the very beginning and found themselves confirmed in their beliefs. As impact CMT is the predominant theme, it is located in the middle of the map and overlaps the other two themes. Within each theme, the three subthemes have different transparencies, depending on the importance (transparent less important). The longitudinal timeline at the bottom shows when certain themes and sub-themes developed and how they changed over time, got more or less important (change in transparency). The pointing arrows show a direct influence from one sub-theme to another one, while the dashed lines show a connection or interaction between two sub-themes. Overlapping sub-themes indicate that these two sub-themes are linked to and influence each other.

#### 3.2.1. Impact of Creative Music Therapy: (a) Positive Effect on Infant (b) Facilitating Parental Relaxation and Well-Being (c) Facilitating Bonding

The parents described that CMT had *a positive effect on their infants,* that they relaxed and stabilized during and after CMT and displayed positive reactions, e.g., smiling, finger movements, and eyes opening. Some parents emphasized a general, long-lasting, and strong effect that not only occurred once but throughout all the sessions, e.g., by using intensifiers like “every time” and “much”, as displayed in the example below, to underline the value of the effect.

*That the children were much calmer every time during music therapy. (I: Yes) They were much calmer during music therapy, afterward, and also when you had them outside or talked to them.*

(5M, 7)

*… and also every reaction from L. [name of infant]. The smallest grin, I would say, or eye movements or whatever.*

(3F (M), 10)

They were fascinated that their infants benefitted from CMT even when they were sleeping and appreciated that their infant had not been awake for the intervention. They underline the social-emotional support for their infant and themselves by outlining that CMT was provided in an individualized, personal interaction. Their infants could experience nurturing contact even when the parents could not be around, which, in turn, decreased the parents’ worries about leaving their child alone in the NICU when they had to leave the hospital; this overlaps with the sub-theme 2c *facilitating parental well-being* (Figure 1).

*Maybe the child knew that it wasn’t alone and someone was there for it.*

(5M, 43)

*And that was nice for me too—to know that even when I’m not there, he also has music therapy and he gets, uh, something.*

(1M, 17)

The parents expressed that CMT might have a longitudinal positive effect on the child’s well-being, musicality, social-emotional, and neurobehavioral development. They appreciated the nurturing, interactive, comfort-giving character of CMT to facilitate basic trust-building and security from the very beginning. However, they expressed these assumptions with more caution, as seen in semantic expressions below such as “I think” and the diminishing color intensity in Figure 1.

*And with the music therapy, I think he has learned that people who engage positively with him, that he can also trust them. So, I think his basic trust is greater than that of a child who was born at term and only trusts his own mother.*

(1M, 17)

*I think she enjoyed it, she still really likes music. Thus, I think whenever there’s music she likes it.*

(4M, 46)

The parents often mentioned the (surprisingly) beneficial effect on the infant in the same breath as the positive effect on themselves. When they felt CMT with all their senses and experienced that their infant relaxed, they could relax as well, indicating that the *positive effect on their infants* resulted in *parental well-being* as the most significant influencing factor for parental well-being.

*Just as I said, that was a (really medical?) but also the soul somehow, the whole thing and also the reactions of L. [name of infant] and also our own, we were surprised.*

(3F (M), 54)

CMT facilitated a calm atmosphere and distraction from worries in the busy and noisy NICU environment. The parents associated CMT with relaxation and reducing stress when they usually felt stressed and worried. They describe the music sessions as consciously experienced quality time, moments of meeting for both infants and parents. However, they indicate that these therapeutic effects for the parents might only work when the parents are capable of engaging, interlinking with the subtheme 2c: *recommendation as a complementary therapy.*

*… And in that sense, it [CMT] is also something that takes the stress away from you. (I: Ok) So you are often in fear and stressed and worried and so on and this music therapy, I think, is not just one for the child, but also one for the parents, who commit to it.*

(1M, 29)

The parents further describe CMT as therapy for themselves, as a place where they could express their emotions without words and where their emotions were given space without judgment. They felt happy and satisfied since they could engage by themselves and experience self-efficacy and parental competency no matter how exactly they engaged. Some parents joined in singing during the therapy, while others sang or spoke to the infants with greater awareness when alone and continued singing at home.

*For me, it was also very emotional yes. … I cried a lot, of course, also during this music therapy … you are calmed down with this music and when the tears start to fall. So, I often cried a lot. And I found it great, of course, that I was not, always asked: “Hey, are you okay?”*

(6M, 69)

*It always made me happy. And also satisfied, because I knew I could do something.*

(1M, 29)

However, one mother pointed out that, particularly at the very beginning, when her infant was very fragile, CMT could only relax her to some extent during the therapy but not in general. During this acute traumatic phase, the daily health status of her infant mainly determined how stressed or relaxed she felt, underlining again how closely the infant’s and parents’ well-being is interconnected.

*… maybe I relaxed a bit more … I don’t know I think you’re in, you’re in such a traumatic situation so I’m not sure you can … I mean, for me it was super nice but I observed even more if she had eaten that day or if she had, if they had to intubate her again … So there were more medical impacts which had a bigger impact on if I was relaxed or not …*

(4M, 61–64)

Closely linked to the positive effects on the infant and the parents themselves is the third subtheme *facilitating bonding*. The parents reported that CMT facilitated, supported, and intensified parental bonding through sensing and adapting to the infant’s breathing rhythm and being positively influenced by the emotional content of the music. They experienced moments of connection and intimacy, indicating that CMT acted as an intensifier. The parents mention that they felt supported by CMT to adapt and tailor emotionally toward their infant and be sensitive to their infant’s clues. They experienced the use of personalized, culturally sensitive music and the multi-sensory experience in CMT of sensing, hearing, seeing, and feeling their infant as helpful to connect emotionally. They even compared CMT to the intimacy and bonding process during breastfeeding and that CMT could act as a surrogate for breastfeeding bonding moments, particularly when breastfeeding is not possible for the mothers.

*And this moment of bonding, through this sharing, well, humming and breathing, attuning to the breath was a very important connection for me, which perhaps also replaces this breastfeeding a bit, which a mother normally has.*

(1M, 15)

*It doesn’t really matter because really with the singing and humming, the parents’ voice and maybe also the oscillation, the vibrations and if the child lies on the skin, then additionally a bond will be established.*

(2F, 23)

#### 3.2.2. Attitude toward CMT: (a) Open-Minded before CMT (b) Value of Music in Daily Life (c) Recommendation as a Complementary Therapy

The overall view of and *attitude toward CMT* developed from an open-minded but skeptical attitude to an appreciation interlinking with the first theme of the positive impact of CMT on the infants, the parents, and the bonding process. The attitude trajectory culminated in a general *recommendation as a complementary therapy* for preterm infants and their parents during and after hospitalization. Throughout the experience of CMT in the NICU, previously reticent and skeptical parental attitudes transformed into a positive to enthusiastic opinion.

*The attitude was a bit reserved, I guess. We had our concerns, especially because of the time. And because there isn’t very much experience with this music therapy. We knew that it was something new but we didn’t expect too much, honestly. (I: Yes) In the beginning.*

(3F (M), 16)

*I don’t see any risk with it so I think you should definitely do it.*

(4M, 24)

*And uhm, for us that was the best decision.*

(6M, 111)

All parents reported a significant *value of music in daily* life. They described music as a central part of the children’s and their own life, accompanying their routines. They mentioned using music to release and or express emotions and feelings and observed these mechanisms in their children as well. They continued to sing for and with their infants, and for some parents, the meaning of music changed from just listening and enjoying to proactively making music and consciously using music to regulate and cope.

*… that has also shown me a lot that you can actually always incorporate music into life. So we sing a lot … it’s unbelievable how much my son also sings and whenever he is stressed somehow ( ) Monday morning he has to go to school, then we start singing.*

(1M, 43)

However, the parents emphasized the value of a trained, professional, emphatic, and calm music therapist. They appreciated the continuous transparent, individualized information about the aims, theories, and methods in CMT and the accompanying supporting, therapeutic conversations while being empowered in their own parental caregiver role.

*And of course, she explained it to me in detail, what she did with the baby, how it affects the child, how I can also contribute myself. And that was an insanely great support that we got there…And there were just insanely great conversations with her.*

(1M, 13–19)

It appeared, that the parents’ attitudes toward specific psycho-social, medical and nursing support varied widely. Some parents appreciated the comprehensive care, responsiveness, and support by doctors, nurses, psychologists, chaplains, and the music therapist. In contrast, other parents explicitly stated that they did not want to have or continue psychological support from psychologists. One mother even felt assaulted, particularly by the answer of a professional who claimed to know how she was feeling.

*I mean I was asked, probably like all mothers, who have had a child so early [sighing] to go to the psychologist. I personally don’t think much of it. I also had a conversation with the psychologist, which for me (rather?), yes…If a lady who is quite a bit younger than me and doesn’t have a child, such a person can’t tell me or can’t [sighs] know how I feel. Nobody can.*

(6 M, 85)

While appreciating CMT, all parents wished for a calmer NICU environment with less stressful noise by alarms, machines, and busy staff. Some parents recommended CMT for all families in the NICU instead of only a few and would welcome the possibility for sharing with other parents. Some parents recommend a music follow-up program after hospital discharge.

*And all these alarms through this intensive care unit …it’s incredibly stressful to hear such an alarm all the time.*

(1M, 13)

*For me, it was all very positive (I: Ok) and you could say I, I thought it was a pity that not all children had access to the music therapy.*

(4M, 16)

#### 3.2.3. Child Development: (a) Developmental Delay (b) Follow-Up Examinations Helpful against Anxiety (c) Overall Healthy Infant Development

Overall, the parents are very grateful for their children’s development and describe them as healthy and happy. Interestingly, some parents concurrently report difficulties such as concentration issues, shyness, and developmental delay, and are ambivalent about interpreting them as personality traits or evident after-effects of the extremely premature birth.

So, has *maybe a slight concentration weakness but that’s actually only (…), not necessarily due to the premature birth I find.

(3F (M), 4)

The parents mention that they had been anxious about their infant’s development. On the one hand, the need to go to the follow-up examinations stressed them, but on the other, after getting positive follow-up results, the examinations reassured them not to worry anymore, resulting in a generally favorable judgment of the follow-up exams as helpful and supporting.

*Yes, it’s like concerns. And so, it was, confirmation after this examination. Then you had like a confirmation that it works, everything is good*.

(3F (M), 66)

Moreover, all parents were hopeful and optimistic that their infants will catch up with their developmental delays. They underline their deep gratitude and great admiration for their children. With pride, as expressed for example by the doubled use of the intensifying adjective “very” in the example below, they emphasize how healthy, happy, and eager to learn the children are. They express how energetic and eager they are, growing and doing well.

*And health-wise I can say, for the fact that she was born so early. She is very rarely, very rarely ill.*

(6M, 19)

*However, this has another level, a deeper level. Also, a thankfulness, that V. [name of infant] is healthy*.

(2F, 67)

**Figure 3:**
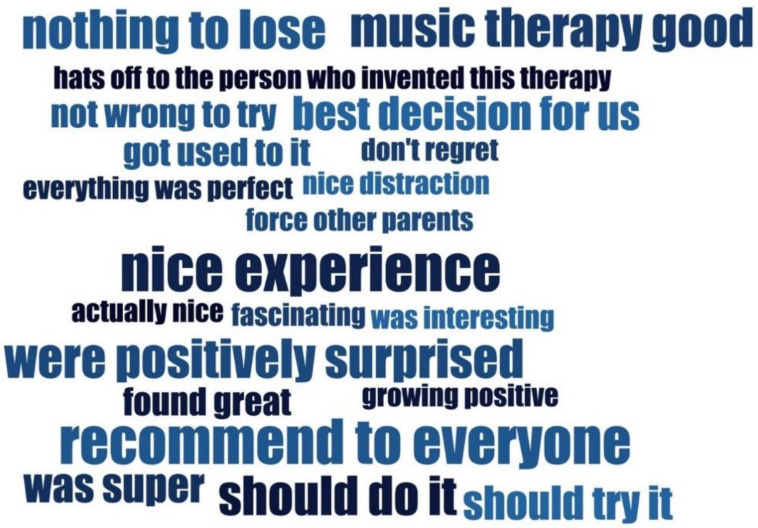
Code Cloud Parental Recommendations.

Finally, all parents recommended CMT as a complementary therapy in neonatal care for its emotional-social value while underlining the superior role of intensive care medicine for survival, as expressed in the example below and visualized with the code cloud (Figure 3).

*So I think both must complement each other. So the purely medical measures are of course enormously important, because, the child would not be able to survive by itself. So it needs 24-h care and also that, the right devices, so that it works at all. And that is very clear. And music therapy is just more on the emotional level. So the medical that is necessary can be complemented …*

(1M, 25)

Code cloud created with MAXQDA 2020 program; sub-codes from the code positive attitude toward music therapy are illustrated as a code cloud, in which the size of each sub-code and saturation correlates with the number of coded segments within the sub-code.

## 4. Discussion

This qualitative inquiry is the first study that has aimed to explore the parental retrospective perspective and experience of CMT with extreme premature infants during neonatal hospitalization and its possible influence on their attitude toward music in daily life and child development during the childhood trajectory. Three main themes were identified: (1) a positive impact of CMT on the infant, the parents, and the bonding, (2) a developing attitude toward CMT from being open-minded to strongly recommending it, and (3) the parental perspective of an overall healthy child development with variations of unique experienced developmental delays over time.

### 4.1. Impact of CMT

In general, it was remarkable how detailed the parents’ recollection was of the time in the NICU seven years ago. The parents described how exhausting and emotionally intense this time was while underlining the positive, formative impact of CMT on their infants, themselves, and their bonding in the stressful NICU setting and beyond. The subthemes of the positive impact of CMT on their infant are in line with previous studies concerning relaxation, stabilization [27,28,42], and stimulation [23,43]. Supporting the results of several qualitative studies [26,44,45], the parents particularly valued that the therapy approach was needs-oriented, smooth, gentle, nurturing, individualized and interactive: a “therapy for the soul” as one mother expressed it. The results emulate the findings of a previous qualitative study of CMT [20,46], suggesting that the finely attuned and entrained infant-directed singing in CMT engages even preterm infants in communicative musicality, supporting self-regulation, pacifying, and stimulation without being overwhelming.

Interestingly, not only physical presence and involvement decreased parental distress caused by limited access to their infant but the knowledge that their infant could experience nurturing contact by a trusted, known person in their absence as well. Particularly in situations and settings where rooming-in is not possible, or parents have limited time to visit their infant, approaches that bridge the gap may be helpful. However, as Shoemark (2011) indicates, when the music therapy is provided without the parents, the therapist has to be sensitive not to act as a surrogate for the parents, but rather to function as an additional source of shared communicative musicality and facilitator for the infant’s social development. The suggested longitudinal beneficial effects on the child’s well-being, musicality, and social-emotional development cannot be confirmed yet since evidence on long-term effects of music therapy, though promising, is still in progress [31,47].

The increase in parental well-being and bonding by relaxation, distraction from worries, empowerment, experienced joy, self-efficacy, parental sensitivity, and closeness with their infant is consistent with previous literature [26,44,45,48]. Notably, we identified similar themes in the context of even a few joined parent-infant sessions of the current data set. In the later study of CMT, where we could show a positive effect on parental anxiety levels, depressive symptoms, adaption, and bonding [26], the parents participated in around 50% of the sessions during skin-to-skin care. In contrast, in the current study, some parents were only able to join a few CMT sessions due to their unavailability or limited overlapping times with the music therapist in the CMT implementation phase. Indeed, music therapy programs, such as “Time Together” [49], with only one parent-infant session, are associated with increased parental self-efficacy [49]. A meta-analysis suggests that few sessions of psycho-educational programs delivered early in infancy are most effective when focusing on parental sensitivity and thereby most effective in supporting attachment and bonding [50] such as that anticipated when using CMT to enhance parental sensitivity [26,44]. However, in our data set, a mother who received only three sessions noticed that under stressful and traumatizing circumstances, CMT could not relax her, indicating that more complex courses of prematurity may need a therapeutic process of several accompanying joint sessions to decrease anxiety levels in the parents as outlined in Kehl et al. [26]. Moreover, all parents reported that they are doing well with no indications for longitudinal psychological distress in contrast to study results on remaining long-term parenting stress of parents with similar inclusion criteria [13,51].

### 4.2. Child Development from the Parents’ Perspective

The positive impact of CMT is strongly interrelated and dependent on the other two main topics of the attitude toward CMT and child development constantly evolving on an individual basis. Like the Health Change Trajectory Model [52], acute crisis or positive health results influenced the experienced extent of the positive effect of CMT and the individual perception of the infant’s health and development during the trajectory from neonatology to school age. The overall positive parental attitude is remarkable, expressing gratitude to pride about their children’s development. Parents describe their children as healthy and happy, although some mention developmental issues in the same breath. Our results mirror the findings of a large mixed-method inquiry with very preterm infants in which the researchers expected that the parents would report mostly negative concerns with 18 months, but in fact, 26.8% of the parents reported only positive aspects, including happiness as one of the main topics [53]. One reason may be that the worries about their infant’s survival during the neonatal period and the overall confrontation with potential risks and developmental problems facilitates gratitude and a shift toward a more positive and humanist perspective [53,54]. Parental unique perceptions, attitudes, feelings, and coping mechanisms may change over time, referred to as posttraumatic growth [55] or positive transformation, as so well put by Janvier et al. [54].

Family-integrating therapeutic programs, such as CMT, may support positive transformation through its core principles of resource and needs-orientation, positive reinforcement, responsiveness, capacity building, culturally sensitive empowerment, and experienced moments of meeting in a safe, nurturing place with creativity and flexibility at the very beginning [17]. All parents reported that music continued to be a central part of their family life, with some parents underlining the positive transformation in the meaning of music from just listening and enjoying to proactively making music and using it consciously to regulate and cope. While in first studies shortly after discharge parents report that they continue singing at home, such a longitudinal influence is a new result and may underline the formative and empowering character of early family-integrating therapeutic approaches such as CMT [26,48].

### 4.3. CMT Recommendation as a Complementary Therapy

All parents were open-minded before CMT started, but the attitude toward CMT varied from skeptical, hesitant, or reticent to neutral to slightly interested. In contrast, now, seven years after the implementation period, the parents and the neonatal team often proactively request CMT, indicating successful implementation in the NICU of the University Hospital Zurich [19]. Following research in implementation science, demonstrating the effectiveness of a clinical innovation such as CMT is not sufficient for successful implementation [56]. Barriers and facilitators have to be identified and addressed as well, suggesting that the positive experience of CMT on the part of parents and the NICU staff may act as a facilitator for implementing CMT. Indeed, despite various skeptical parental attitudes toward CMT before therapy commenced in the study cohort, all parents without exception recommended CMT as a complementary therapy in neonatal care for all infants, without having to be asked directly about it. This overall recommendation is of value since, to our knowledge, an explicit parental recommendation of music therapy as a complementary therapy for preterm infants and their caregivers is a new result, although quantitative and qualitative studies underline various positive effects on the infants and their parents [27,42,48]. Future research may further examine the parents’ perspective with a larger sample size to explore if and how to provide complementary therapies such as CMT in neonatal care, including challenges and limitations. Indeed, health service organizations call for clinical governance that involves patients and their representatives/caregivers by patient/caregiver-led and patient/caregiver-responsive decision making [57,58].

The parents go even further by recommending CMT for all infants in the NICU instead of only for the families who participate in a study or are referred by the neonatal team most in need because of limited music therapy service resources. These findings call for extending music therapy services and or complementary, ecologically appropriate approaches such as “Time Together” by Shoemark [59]. Time Together is a strength-based program where a music therapist provides one personalized session inclusive of a written booklet focusing on parental capacity and self-efficacy building by knowledge sharing and coaching on parental vocal interactive competencies. The approach supports the current CMT clinical practice where these days, all parents in neonatal care receive an international lullaby book [60] with written information about why and how to hum, speak and sing to their infant, to empower and motivate the parents to get involved in vocal, nurturing interaction. This approach is based on the theories of empowerment [61] by using peers, other parents of preterm infants to discover and use the parents‘ innate capability for parent-infant relationship and capacity building supplemented by further peer-led sharing of experiences and knowledge of an open-access web platform, called amiamusica (https://amiamusica.ch (accessed on 31 May 2021)). Interestingly, one of the mothers interviewed called explicitly for such a platform enabling her to share her personal experiences with peers who entirely understand her perspective. Indeed, engaging in social media sharing has been reported as a helpful coping strategy by several authors [62,63].

Some parents called for music therapy to be continued after discharge, as is currently being implemented and evaluated with a multi-center international study where music therapy home services bridge the transition from hospitalization to home [47]. In general, many parents experience the transition to the home phase as a challenging time due to feelings of unpreparedness, increased needs for knowledge, and poor coordination of discharge services [64]. This is why various home-based preventive care programs with beneficial long-term benefits on cognitive and motor outcomes in infants have been implemented around the world [65]. In Switzerland, the piloted transition to home model provides continuing interdisciplinary support for the child and family, including caregiving, music therapy, physical therapy, and psychological support [66]. Interestingly, a meta-analysis indicates that programs which were provided both in the home and in the hospital/preschool setting are associated with the most beneficial effects, indicating that there should be multiple locations and settings, e.g., music therapy home services and music parent-infant groups, to meet the varying needs of the families, to enable meeting and sharing with other families or to receive services in the home setting [15]. However, some parents may be happy to be left alone without an imposed schedule for feeding, diaper changing, and therapy appointments and wish to settle by themselves as a typical family with the desire for normality. This result is in line with previous results describing that some parents long to let go of their premature parental identity, even if they had EP infants, and that their need for support varied greatly [62].

While recommending CMT, one mother suggested that the will and sensitivity of parents may be an essential factor for CMT to work. Another mother pointed out that CMT could not relax her when she was too stressed and occupied with the infant’s severe acute health conditions. Some parents underlined the superior role of medicine in intensive care medicine. These limitations are in line with the overall attitude of CMT with its “subordinated and complementary role in the lifesaving priority setting of medical treatment and intensive care…and that CMT with parental integration can only occur when the parents are present and willing to participate” [19]. High sensitivity and overall responsiveness to the inter-weaving and constantly-changing needs of the infant, parents, and the NICU environment are needed for daily clinical decision-making, which may entail rescheduling a therapy or allowing space for other therapies and/or professionals as identified in a former qualitative inquiry of CMT [44,67]. Interdisciplinary psycho-social support for parents to choose their favorite therapy seems optimal since some parents preferred or declined specific therapies, while others appreciated pastoral work, psychological and music therapy services to the same extent. Indeed, best practice guidelines recommend a multi-layered approach for parents in the NICU in order to meet individual varying needs [51].

### 4.4. Limitations and Strengths

A limiting factor of the current study is that the sample size was determined by the prospective feasibility study inclusion rather than by qualitative considerations. The drop-out rate six years later at the time of the interview was relatively high, with 40% of drop-outs attributable to inaccessibility after such a long lifespan. However, the sample might still be sufficient given the large sample variation in parental characterization, such as high cultural and socioeconomic diversity within a sample of parents who all had extremely born preterm infants treated with CMT in the same hospital setting [68]. This is particularly true since the sample size was large enough to allow for a rich and new understanding of the longitudinal perspective of the parents but small enough to facilitate an in-depth case-oriented analysis [69]. Qualitative interviews with more fathers would have enabled us to gain a more explicit understanding of the perspective of the fathers [70].

One strength of our study is that an unknown researcher interviewed the parents when they were no longer dependent on the research team and/or institution for therapeutic, medical, or other caretaking or follow-up programs of their infant. Furthermore, a dual deductive–inductive approach was possible since one researcher had no prior experience or knowledge of CMT while the other researcher is an expert in the research field, realizing a hybrid process of inductive and deductive thematic analysis to interpret raw data [34]. Additionally, cultural diversity in the sample was feasible since the interviewing researcher was fluent in three different languages. Another strength concerns the continuing discussions and double-checking of accuracy in all stages of the qualitative inquiry in data acquisition, transcription, analysis, and reporting through continuing close supervision.

## 5. Conclusions

Seven years after giving birth, the parents of our qualitative inquiry express the positive, formative impact of CMT on their extremely premature-born infants, themselves, and bonding in the stressful NICU setting and beyond. Their experiences culminated in general recommendations of CMT for other families, although these varied in unique meaningful details. CMT may empower positive transformation in the parents through individualized, need and resource-based early nurturing musical interactions, capacity building, and positive reinforcement. More research is needed on potential challenges and limitations of music therapy from the parents’ perspective, e.g., using quantitative online surveys. Implementation science is warranted to explore the facilitating and limiting mechanisms of the clinically innovative CMT program. Further qualitative and quantitative studies examining parental experiences and mental health as well as the social-emotional, neurobehavioral development and quality of life in the infants across the lifespan may help to identify potentially modifiable factors for improving health care in this vulnerable group with early individualized approaches such as CMT.

## Figures and Tables

**Table 1:** Sample Characteristics.

Participants (*n*)	Parents (6: 4 Mothers & 2 Fathers)	Infants (7)
Interview partner nationality (*n*)		
Swiss	*2*	
German	1	
Danish	*1*	
Slovenia	1	
Kosovo	1	
Interview partner educational qualification		
(% (*n*))		
Compulsory education	17 (1)	
Apprenticeship	33 (2)	
University degree	50 (3)	
Primigravida (% (*n*))	33 (2)	
Primiparous (% (*n*))	66 (4)	
Twins (% (*n*))		29 (2)
Male infants (% (*n*))		29 (2)
Gestational age at birth (weeks) (median (range))		25.57 (24–27)
Birth weight (g) (median (range))		854.29 (610–1070)
Birth size (cm) (median (range))		33.43 (30–38)
Apgar score (10 min) (median (range))		6.2857 (4–8)
Chorioamnionitis (% (*n*))		42.86% (3)
ROP (% (*n*))		28.57% (2)
BPD (% (*n*))		14.29% (1)
Intubation days (median (range))		5 (0–13)
Cerebral haemorrhage (% (*n*))		0 (0)
Ventricular dilatation (% (*n*))		0 (0)
Sepsis (% (*n*))		0 (0)
NEC (% (*n*))		0 (0)
Day of discharge (median (range))		88.57 (58–117)
Weight at discharge (median (range))		3211.43 (2540–4480)

**Figure A1:**
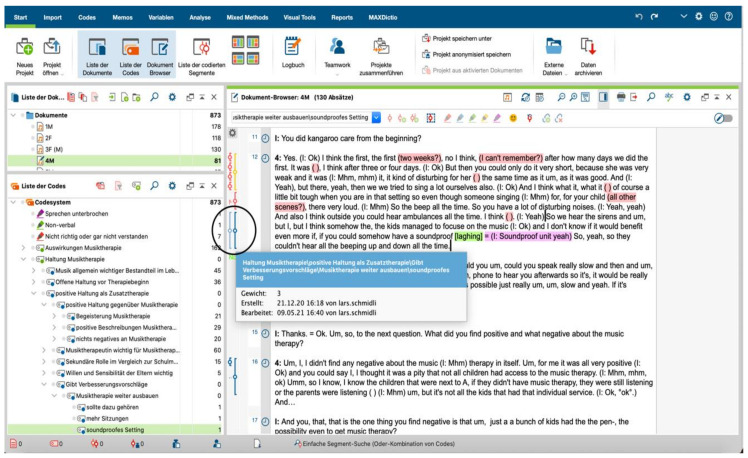
Screenshot of MAXQDA 2020 Program.

**Figure A2:**
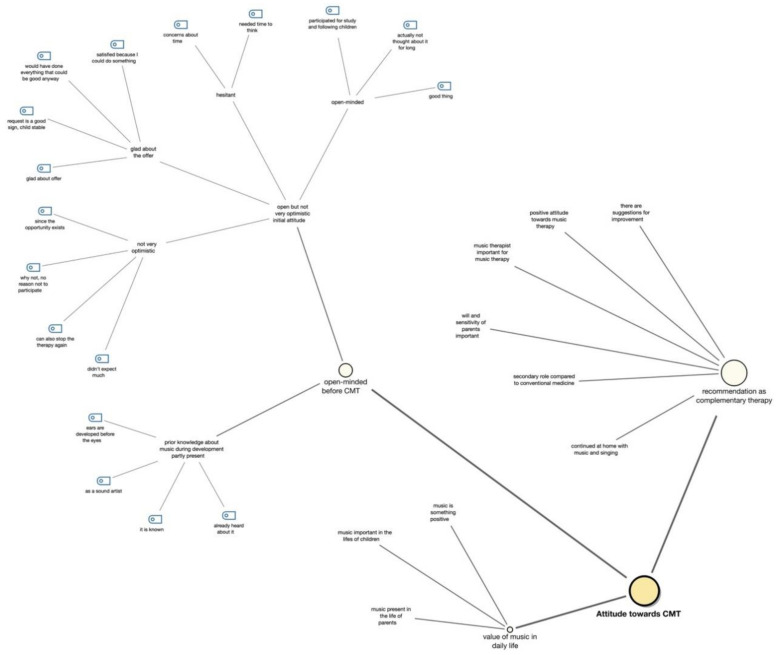
Detailed Coding Tree.

## Data Availability

Additional data can be requested from the corresponding author.

## Appendix A. Semi-Structured Interview Guide

Semi-Structured Interview-Guide CMT for Parents of Extremely Preterm Infants.

Around six years ago, you and your child/children participated in a study on music therapy. Now, I would like to find out how you experienced this time in the neonatology unit and how you subjectively felt about music therapy. I would like to ask you a few questions about this. Of course, all your responses and comments will be treated confidentially.

### Appendix A.1. Introductory Questions

How are you and how is your child?

What do you remember from your time in neonatology?

### Appendix A.2. Main Questions

1. What do you remember from the music therapy?
2. What did you find positive and what negative about the music therapy?
3. What was your attitude toward music therapy when you were first informed about it and how did that attitude develop during the therapy process?
4. How would you rate music therapy in terms of benefits and risks? And in your opinion, how can this be compared with purely medical procedures?
5. How did you experience your time in neonatology in general and how did you feel during and after the therapy sessions?
6. How did you see your role during music therapy and did you take an active part?
  - If actively involved: Did you observe anything in your child during or after music therapy?
7. How would you describe the bond with your child throughout the therapy process?
  - Has the bond with your child changed due to a change in its emotional state and the role taken in therapy?
8. How was the interaction with the music therapist?
9. What place does music have in your life?
10. Had the monochord already been used for therapy at the time? If yes: How did you feel the vibrations from the monochord during kangarooing?
11. What were your thoughts before the neurological follow up exams after two and five years?
  - Has your child already started school? If so, how did you feel about it?
12. Music therapy is offered to families with a prematurely born baby and they would like to hear your opinion about it. What would you tell them?

### Appendix A.3. Final Question

We’ve already reached the last question. Is there anything else that is important to you and that has not yet been addressed or do you have any questions?

Thank you very much for taking the time.

## Appendix B

Purple: speaker got interrupted; green: nonverbal expression; red: did not (fully) understand. Inside the black circle are two blue markings for a coded segment (coded segment also marked with two black lines in the transcript) in the transcript on the right. These two markings stand for different sub-codes (the brighter one codes for soundproof setting, seen at the bottom left in the “list of codes”).

## Appendix C

Code cloud created with MAXQDA 2020 program; sub-codes from the code positive attitude toward music therapy are illustrated as a code cloud, in which the size of each sub-code correlates with the number of coded segments within the sub-code.
